# Supplementary material for: Bacterial profile and antimicrobial susceptibility patterns in cancer patients
Source: PLoS One. 2022 Apr 15;17(4):e0266919. doi: 10.1371/journal.pone.0266919 (PMC9012398; doi:10.1371/journal.pone.0266919)
Supplement: S1 Table — (DOCX) [file pone.0266919.s001.docx]

**S1 Table. The AST interpretation chart (extracted from CLSI, 2021).**

AST for Enterobacteriaceae

| S. No | Drug Name | Susceptible (mm) | Intermediate (mm) | Resistant  (mm) | Remark |
| --- | --- | --- | --- | --- | --- |
| 1 | Chloramphenicol | >18 | 13-17 | < 12 |  |
| 2 | Azithromycin | >13 |  | < 12 |  |
| 3 | Cotrimoxazole | >16 | 11-15 | < 10 |  |
| 4 | Tetracycline | >15 | 12-14 | < 11 |  |
| 5 | Doxycycline | >14 | 11-13 | < 10 |  |
| 6 | Gentamicin | >15 | 13-14 | < 12 |  |
| 7 | Amoxicillin-clavulanic acid | >25 |  | < 18 |  |
| 8 | Ceftriaxone | >23 | 20-22 | < 19 |  |
| 9 | Ciprofloxacin | >26 | 22-25 | < 21 |  |
| 10 | Ceftazidime | >21 | 18-20 | < 17 |  |
| 11 | Ampicillin | >17 |  | < 13 |  |

AST for *S. aureus*

| S. No | Drug Name | Susceptible (mm) | Intermediate (mm) | Resistant (mm) | Remark |
| --- | --- | --- | --- | --- | --- |
| 1 | Gentamycin | >15 | 13-14 | <12 |  |
| 2 | Penicillin | > 29 |  | <28 |  |
| 3 | Tetracycline | >19 | 15-18 | <14 |  |
| 4 | Cefoxitin | >21 |  | < 21 |  |

AST for *P. aeruginosa*

| S. No | Drug Name | Susceptible (mm) | Intermediate (mm) | Resistant (mm) | Remark |
| --- | --- | --- | --- | --- | --- |
| 1 | Gentamicin | >15 | 13-14 | < 12 |  |
| 2 | Piperacillin | >21 | 15-20 | < 14 |  |
| 3 | Ciprofloxacin | >25 | 19-24 | < 18 |  |
| 4 | Ceftazidime | >18 | 15-17 | < 14 |  |
| 5 | Meropenem | >19 | 16-18 | < 15 |  |
| 6 | Tobramycine | >15 | 13-14 | < 12 |  |
| 7 | Amikacin | >17 | 15-16 | < 14 |  |
