# Supplementary material for: Bacterial profile and antimicrobial susceptibility patterns in cancer patients
Source: PLoS One. 2022 Apr 15;17(4):e0266919. doi: 10.1371/journal.pone.0266919 (PMC9012398; doi:10.1371/journal.pone.0266919)
Supplement: S2 Table — (DOCX) [file pone.0266919.s002.docx]

**S2 Table. Antimicrobial susceptibility pattern collection form.**

| Code number | | |  | | | | | | |
| --- | --- | --- | --- | --- | --- | --- | --- | --- | --- |
| Hospital card number | | |  | | | | | | |
| Date of specimen collection | | |  | | | | | | |
| Health Organization | | |  | | | | | | |
| S. No |  | | Code | | | | | | Response |
| 1. | Bacterial isolates | | 1*= S. aureus*, 2 *= K. pneumoniae*, 3 = *P. aeruginosa,* 4 = *A. baumannii*, 5 = *E. coli* | | | | | |  |
| 3. Antimicrobial susceptibility pattern | | | | | | | | | |
| Antibiotics | | AST results (CLSI, 2021): Zone diameter breakpoints nearest to whole millimetre | | | | | | | |
|  |  | Content | | S | I | R | Reading (mm) | Interpretation | |
| Ceftriaxone | | 30µg | |  |  |  |  |  | |
| Ciprofloxacin | | 5µg | |  |  |  |  |  | |
| Tetracycline | | 30µg | |  |  |  |  |  | |
| Tobramycine | | 10µg | |  |  |  |  |  | |
| Amikacin | | 30µg | |  |  |  |  |  | |
| Meropenem | | 10µg | |  |  |  |  |  | |
| Penicillin | | 10µg | |  |  |  |  |  | |
| Gentamicin | | 10µg | |  |  |  |  |  | |
| Co-trimoxazole | | 1.25+23.75 μg | |  |  |  |  |  | |
| Ampicillin | | 10µg | |  |  |  |  |  | |
| Augmentin | | 20/10 µg | |  |  |  |  |  | |
| Cefoxitin | | 30µg | |  |  |  |  |  | |
| Piperacillin | | 100 µg | |  |  |  |  |  | |
| Ceftazidime | | 30 µg | |  |  |  |  |  | |
