# Supplementary material for: Bacterial profile and antimicrobial susceptibility patterns in cancer patients
Source: PLoS One. 2022 Apr 15;17(4):e0266919. doi: 10.1371/journal.pone.0266919 (PMC9012398; doi:10.1371/journal.pone.0266919)
Supplement: Attachment — Submitted filename: Response to Reviewers.docx [file pone.0266919.s004.docx]

**To: The Editor of PLOS ONE**

**Title:** Bacterial profile and antimicrobial susceptibility patterns in cancer patients

**Submission ID:** PONE-D-21-38142

Corresponding author: Abiye Tigabu

Thank you for considering our article for revision and we are grateful to the editor and the reviewers for their constructive comments.

**Point by point response to editor comments**

1. Please ensure that your manuscript meets PLOS ONE's style requirements, including those for file naming.

- We revised the manuscript according to PLOS ONE's style requirements.

2. You indicated that you had ethical approval for your study. In your Methods section, please ensure you have also stated whether you obtained consent from parents or guardians of the minors included in the study or whether the research ethics committee or IRB specifically waived the need for their consent.

- A statement that states about “consent from parents or guardians of the minors” added in the revised manuscript.

3. We note that you have stated that you will provide repository information for your data at acceptance. Should your manuscript be accepted for publication, we will hold it until you provide the relevant accession numbers or DOIs necessary to access your data. If you wish to make changes to your Data Availability statement, please describe these changes in your cover letter and we will update your Data Availability statement to reflect the information you provide.

- We declared that all data underlying the findings described fully available, without accession numbers or DOIs necessary to access our data upon publication of the work, and the changes described in the cover letter.

4. We noticed you have some minor occurrence of overlapping text with the following previous publication(s), which needs to be addressed:

- https://bmcresnotes.biomedcentral.com/track/pdf/10.1186/s13104-019-4520-9.pdf

- https://link.springer.com/article/10.1007%2Fs15010-004-3049-5

- https://www.dovepress.com/microbial-spectrum-and-drug-resistance-profile-of-isolates-causing-blo-peer-reviewed-fulltext-article-IDR

The text that needs to be addressed involves the Discussion.

In your revision ensure you cite all your sources (including your own works), and quote or rephrase any duplicated text outside the methods section.

- Overlapping texts reviewed and corrected throughout the revised manuscript and the sources of information are cited.

**Point by point response to Reviewer 1**

**Title**

1. I think the work would benefit from the title that contains the main conclusion of the study (should be derived from the conclusion). Please modify the title.

- The title modified in the revised manuscript accordingly.

**Abstract**

2. The abstract must illustrate the used methods and the most prevalent results (give more hints about methods and results). Besides, rephrase the aim of the work and the main conclusion of your findings.

- The methods used and the most prevalent findings of the study added in the revised manuscript. Additionally, the aim and the conclusion of the work rephrased.

**Introduction**

3. It needs to be more informative?

- Information from several studies added and the introduction rewritten to make informative in the revised manuscript.

4. Give a hint about the virulence factors, different infections caused by *S. aureus*, *K. pneumoniae*, and *Pseudomonas aeruginosa*, and the mechanism of disease occurrence.

- Virulence factors, different infections and disease mechanism caused by *S. aureus*, *K. pneumoniae*, and *P. aeruginosa* added in the revised manuscript.

5. The authors should illustrate the public health importance concerning the emergence of multidrug-resistant (MDR) bacterial pathogens that reflect the necessity of new potent and safe antimicrobial agents. Several studies proved the widespread MDR- bacterial pathogens;

Authors could add the following paragraph:

Multidrug resistance has been increased all over the world that is considered a public health threat. Several recent investigations reported the emergence of multidrug-resistant bacterial pathogens from different origins including humans, birds, cattle, and fish that increase the need for routine application of the antimicrobial susceptibility testing to detect the antibiotic of choice as well as the screening of the emerging MDR strains. You should cite the following valuable studies:

1. PMID: 33177849

2. PMID: 32397408

3. PMID: 32994450

4. PMID: 32497922

5. PMID: 33061472

6. PMID: 33947875

7. PMID: 34445951

8. PMID: 33188216

9. https://doi.org/10.1016/j.aquaculture.2021.737643

10. PMID: 30150182

- The paragraph recommended by the reviewer and the public health importance of MDR bacterial pathogens illustrated in the revised manuscript.

**Material and methods**

6. Illustrate your methods with subtitles.

- Methods stated as subtitles in the revised manuscript.

7. Add this subtitle: Bacterial isolation and identification

- The subtitle bacterial isolation and identification added in the revised manuscript.

8. Discuss in detail the methods of isolation and identification of *S. aureus* and MRSA. Besides, specific references should be added.

- The detail methods of isolation and identification of *S. aureus* and MRSA revised accordingly.

9. Add the company, city, and country of the used bacterial media and reagents that were used in the biochemical identification of isolates. Also, enumerate all used biochemical reactions.

- The company, city, and country name of the used culture media and reagents stated in the revised manuscript.
- Biochemical tests used in study enumerated and corrected in the revised manuscript.

**Antimicrobial susceptibility testing**

10. Illustrate the antimicrobial classes of the tested antimicrobial agents within the text.

- Antimicrobial classes of the tested antimicrobial illustrated in the revised manuscript.

11. The authors are advised to classify the tested isolates to MDR, XDR, and PDR as described by Magiorakos et al.

Magiorakos AP, Srinivasan A, Carey RB, Carmeli Y, Falagas ME, Giske CG, et al. Multidrug-resistant, extensively drug-resistant and pandrug-resistant bacteria: An international expert proposal for interim standard definitions for acquired resistance. Clin Microbiol Infect. 2012; 18:268–81. doi:10.1111/j.1469-0691.2011.03570.x.

- Tested isolates classified to MDR, XDR, and PDR as described by Magiorakos et al.

12. Why did you ignore the detection of antibiotic resistance genes in the recovered isolates? Please use PCR to detect antibiotic resistance genes and virulence genes, followed by gene sequencing if possible. Afterward, the correlation between phenotypic and genotypic multidrug resistance should be performed.

- Due to budget constraints and lack of facility, we were unable to perform molecular analysis such as detection of antibiotic resistance and virulence genes using PCR, and gene sequencing to associate between phenotypic and genotypic multidrug resistance in the recovered isolates and it is included as limitation in the revised manuscript.

13. Add more details about the software used in the statistical analyses.

- Software’s used for statistical analyses added in the revised manuscript.

**Results**

14. Add this subtitle: Phenotypic characteristics of the recovered isolates.

- The subtitle “Phenotypic characteristics of the recovered isolates” added in the revised manuscript.

15. Illustrate in detail the phenotypic characteristics of the recovered isolates, especially, *S. aureus*, *K. pneumoniae*, and *P. aeruginosa*.

- Phenotypic characteristics of *S. aureus*, *K. pneumoniae*, and *P. aeruginosa* included in the revised manuscript.

16. Illustrate in a new table the occurrence of MDR (Multidrug resistance) among the recovered isolates (illustrate the names of the antimicrobial classes and different antibiotics):

No. of strains % Type of resistance

R, MDR, and XDR Phenotypic multidrug resistance

(Antimicrobial classes and different antibiotics). The antibiotic -resistance genes.

- A new table added to illustrate multidrug resistance, No. of strains and % type of resistance in the revised manuscript.

17. Increase the resolution of all figures (it should be 600 dpi).

- The resolution of all figures increased the revised manuscript.

**Discussion**

18. The authors are advised to illustrate the real impact of their findings without repetition of results.

- The real impacts of the findings discussed and the repetition of results removed.

19. Illustrate the different mechanisms of antimicrobial resistance in *S. aureus*, *K. pneumoniae*, and *Pseudomonas aeruginosa*.

- The antimicrobial resistance mechanisms of *S. aureus*, *K. pneumoniae*, and *P. aeruginosa* illustrated in the revised version of the manuscript*.*

**Conclusion**

20. Should be rephrased to be sounded. A real conclusion should focus on the question or claim you articulated in your study, which resolution has been the main objective of your paper?

- The conclusion of the finding rephrased in the revised manuscript.

**Point by point response to Reviewer 2**

**General comments**

1. The current study has a significant impact, but it needs a major revision.

- The manuscript revised in detail throughout the revised manuscript.

2. The manuscript should be revised for grammar mistakes.

- Grammar mistakes and spelling problems corrected in the revised manuscript.

3. Please write the scientific names of bacterial pathogens and genes in the correct form all over the manuscript and in the References section (should be italic).

- Names of bacterial pathogens written correctly throughout the revised manuscript.

4. The title is broad, please modify the title.

- The title modified in the revised manuscript accordingly.

5. Add more details about the used methods and most prevalent results in the abstract.

- Methods used and the most prevalent findings of the study added in the abstract section of the revised manuscript.

**Introduction**

6. Discuss the public health importance of *S. aureus*, *K. pneumoniae*, and *Pseudomonas aeruginosa*, and their virulence determinants.

- Public health importance and virulence determinants of *S. aureus*, K*. pneumoniae*, and *P. aeruginosa* added in revised manuscript.

7. Improve the aim of work.

- The aim of the study improved in the revised manuscript.

**Methods**

8. Explain the methods of isolation and identification in detail??

- Methods of isolation and identification explained in a clear and detail manner.

9. Specific references should be added to all the used methods and techniques.

- References added for the specific methods and techniques used.

10. Antimicrobial susceptibility testing: Add the manufacturing company, city, and country for the used reagents and antimicrobial discs.

- The names of manufacturing company, city, and country of used reagents and antimicrobial discs included in the revised manuscript.

11. PCR based detection of virulence genes and antimicrobial resistance genes in the most prevalent retrieved bacterial species should be carried out if applicable (or addresses this point in the study limitations)

- Due to budget constraints and lack of facility, we were unable to perform molecular analysis such as detection of antibiotic resistance and virulence genes using PCR, and gene sequencing to associate between phenotypic and genotypic multidrug resistance in the recovered isolates and it is included as limitation in the revised manuscript.

**Results**

12. Discuss in detail the phenotypic characters of the recovered isolates: *S. aureus*, *K. pneumoniae*, and *Pseudomonas aeruginosa*.

- Phenotypic characters of *S. aureus*, *K. pneumoniae*, and *P. aeruginosa* presented in detail in result section of the revised manuscript.

13. Increase the resolution of different Figures: Please improve.

- The resolution of figures improved in the revised manuscript.

14. PCR based detection of virulence genes and antimicrobial resistance genes in the most prevalent retrieved bacterial species should be carried out if applicable (or addresses this point in the study limitations)

- Due to budget constraints and lack of facility, we were unable to perform molecular analysis such as detection of antibiotic resistance and virulence genes using PCR, and gene sequencing to associate between phenotypic and genotypic multidrug resistance in the recovered isolates and it is included as limitation in the revised manuscript.

15. The correlation between the phenotypic and genotypic MDR should be performed.

- We were unable to associate the correlation between the phenotypic and genotypic MDR due to resource constraints and it is mentioned as limitation in the manuscript

**Discussion**

16. Please improve.

- The discussion rewritten and improved in the revised manuscript.

17. Please improve the main conclusion of the manuscript.

- The conclusion of the finding rephrased and improved in the revised manuscript.
